# The Humanitarian Situation in Syria: A Snapshot in the Third Year of the Crisis

**DOI:** 10.1371/currents.dis.6b7587562e58cf965bf2d7be36f96de6

**Published:** 2015-03-03

**Authors:** Shannon Doocy, Tefera D. Delbiso, The IOCC/GOPA Study Team, Debarati Guha-Sapir

**Affiliations:** Johns Hopkins Bloomberg School of Public Health, Baltimore, Maryland, United States; Center for Research and Epidemiology in Disasters, Catholic University of Louvain, Brussels, Belgium; International Orthodox Christian Charities (IOCC) and the Greek Orthodox Patriarch of Antioch and All the East (GOPA), Baltimore, Maryland, USA; Center for Research and Epidemiology in Disasters, Catholic University of Louvain, Brussels, Belgium

## Abstract

Between April and June 2014, International Orthodox Christian Charities (IOCC), an International NGO, and the Greek Orthodox Patriarchate of Antioch and All the East (GOPA) conducted a needs assessment of Syrians affected by the crisis with the objective of gaining a better understanding of humanitarian needs and assistance priorities. Findings suggest that interventions that increase access to non-food items, food, medication and education should be prioritized where cost was the primary barrier to accessing goods and services. Cash transfer programs and direct provision of material assistance should be considered, though the most appropriate assistance modality is likely to vary by sector, location and the preferences and prior experience of donors and implementing organizations. Renewed international commitment to funding humanitarian assistance efforts in Syria and neighboring countries where the burden of refugees is greatest is essential from both a human rights perspective and in terms of maintaining stability in the region.

## Introduction

The Syrian conflict has resulted in immense suffering. Violence throughout the country has displaced millions, both within Syria and as refugees, and millions more are affected by the conflict and have unmet needs. The United Nations High Commissioner for Refugees (UNHCR) had registered more than 2.8 million Syrian refugees (as of June 2014) and within Syria, there an estimated 7.6 million internally displaced people (IDPs) and more than 11.5 million people in need of assistance (as of April 2014).^1^^,^ 2 There is little indication the large-scale displacement and humanitarian crisis will end soon.

Between April and June 2014, International Orthodox Christian Charities (IOCC), an International NGO, and the Greek Orthodox Patriarchate of Antioch and All the East (GOPA), providing humanitarian assistance in Syria, conducted a needs assessment of Syrians affected by the crisis. The objective was to enable IOCC and GOPA to better understand humanitarian needs and assistance priorities. Because of the limited data available in Syria, this assessment, which is inclusive of nearly 4,000 households, is a valuable source of information that can contribute to an improved understanding of the impacts of the crisis and unmet humanitarian needs.

## Methods

The assessment was conducted between April and June 2014 in nine governorates of Syria (Aleppo, Al-Hasakeh, As-Sweida, Damascus, Dara’a, Homs, Latakia, Rif Damascus, and Tartous). Given that no recent and accurate nationwide estimates of the displaced population or the population in need of assistance have been available, planning a representative sample in the Syrian context is exceptionally difficult. Furthermore, security and access issues are a persistent and evolving challenge that limited the ability to attain the planned sample. As such, the sample is best viewed as a purposive sample of households in need of assistance in accessible communities, which were predominantly in government controlled areas. Within these governorates, 36 communities/neighborhoods in 19 districts were included (Figure 1). Included communities met the following criteria: 1) no recent needs assessment from other organizations was at the time available; and 2) large numbers of displaced or otherwise conflict -affected families that were perceived as vulnerable, poor or underserved with humanitarian aid were present. Communities were excluded if they met any of the following criteria: 1) the assessment could present a security threat to interviewers or respondents; 2) significant humanitarian assistance was being received; or 3) the community was perceived as affluent.

**Figure:**
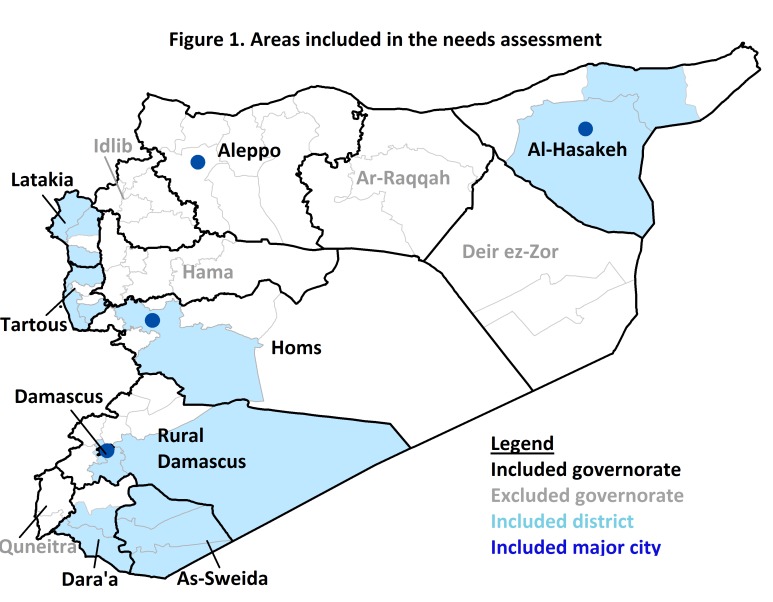

The assessment was intended to sample different types of households in the community. In each location, the planned sample was 30 households (i.e. a 36x30 cluster design). Eligible households included those that were displaced; host families of those displaced; returnees; and those otherwise directly affected by the conflict. For areas with large numbers of families registered with IOCC/GOPA, including those receiving and not receiving humanitarian assistance, a list-based sampling approach was used where households were randomly selected from the IOCC/GOPA database. For areas with few or no registered households, local community leaders were asked to refer the survey team to under-served families. Where possible, multiple sources of information were sought in each community. Lists were combined and cross checked so that families listed multiple times could be prioritized; a random list-based sample of households identified by one community leader was then used to sample the remaining households.

Data was collected using a structured multi-sectoral paper questionnaire developed by IOCC/GOPA. Interviews were conducted by IOCC/GOPA staff based in offices throughout the survey area. Interviewers received training on household selection, interviewing techniques, and the survey tool, and were provided with a survey guide. Interviews were conducted by one or two staff as appropriate, given the context and respondent gender. Interviewers were instructed to invite both the household head and wife to participate if available; if only one was available this individual was interviewed, and if neither was available, the household identified an alternative adult respondent. Potential respondents were informed that participation was voluntary and that no humanitarian assistance or other direct benefit would result from participation in the survey. Interviews were 20-30 minutes in duration.

Data was entered in Google Forms, exported into excel for cleaning and coding, and then imported into SPSS 19 and EpiInfo for analysis. Descriptive statistics are presented for both the needs sample (unweighted) and for the estimated population with unmet needs across the nine governorates surveyed (weighted). Sample weights were derived from the most recent available information on the population with unmet needs (Table 1), and were used to adjust the distribution of the sample.^1^

Permission to conduct the assessment was sought from community leaders prior to approaching households for interviews; local ethical review approval was not required because the primary aim of the assessment was not research. The Johns Hopkins School of Public Health Institutional Review Board (IRB) approved the analysis of the de-identified data set as non-human subjects research.

**Table 1: Distribution of IDPs, Populations in Need, and the Assessment Sample*:** 

	Internally Displaced		In Need		Assessment Sample**		
	N	%	N	%	N	%	Weight
Aleppo	1,735,000	22.8%	2,456,000	22.0%	131	3.4%	4.846
Al-Hasakeh	327,000	4.3%	654,000	5.9%	547	14.2%	0.309
Ar-Raqqa	308,000	4.0%	507,000	4.5%	-	-	-
As-Sweida	52,000	0.7%	53,000	0.5%	507	13.1%	0.027
Damascus	318,000	4.2%	620,000	5.6%	329	8.5%	0.487
Dara’a	372,000	4.9%	399,000	3.6%	507	13.1%	0.203
Deir-ez-Zor	517,000	6.8%	835,000	7.5%	-	-	-
Hama	423,000	5.6%	836,000	7.5%	-	-	-
Homs	588,000	7.7%	680,000	6.1%	318	8.2%	0.553
Idleb	724,000	9.5%	948,000	8.5%	-	-	-
Latakia	908,000	11.9%	921,000	8.3%	373	9.7%	0.638
Quneitra	78,000	1.0%	96,000	0.9%	-	-	-
Rif Damascus	762,000	10.0%	1,600,000	14.3%	595	15.4%	0.695
Tartous	500,000	6.6%	550,000	4.9%	562	14.6%	0.253
National Total	7,612,000	100.0%	11,155,000	100.0%	3,869	100.0%	---
*Internally displaced and population in need estimates as of April, 2014							
**weighting determined based on governorate level estimates of population in need							

## Results

The needs assessment included 3930 households; respondents were predominantly male (54.7%, CI: 52.4-56.8) and averaged 43 years of age (range 14-89); a majority of respondents indicated they were the household head (70.6%, CI: 68.5-72.6%). Median household size was six (range 1-35); nearly all households (83.5%, CI: 81.7-85.3) had children <18 years (median=3) and a majority of households (65.9%, CI: 63.6-68.1) had children <5 years of age (median=2). The vast majority (82.4%, CI: 80.7-84.1) of households included in the assessment were displaced; among displaced households, 72.3% (CI: 70.0-74.5) had been displaced for more than a year; 13.7% (CI: 12.1-15.5) had been displaced for between six months and a year and 13.9% (CI: 12.3-15.7)were displaced for less than six months.

Unmet needs were aggregated by sector to understand which sectors were perceived as priority areas for humanitarian assistance. A household reporting any specific need within the sector as one of their top five priorities for aid was classified as having an unmet need within that sector. The sector with highest reported level of unmet needs was non-food items (89%); this followed by health (51%), food (50%), shelter (43%), livelihoods (33%) and education (25%) (Figure 2).‡ A substantial proportion, 41.5% (CI: 39.3-43.8), of households were receiving non-food item (NFI) assistance. Despite receipt of assistance, almost all households (95.9%, CI: 94.9-96.7) reported having unmet NFI needs, indicating that continued distribution of NFIs is essential. The most frequently reported unmet NFI needs included hygiene and personal items (64%), clothing (52%), bedding (37%) and cooking utensils (26%).‡

**Figure:**
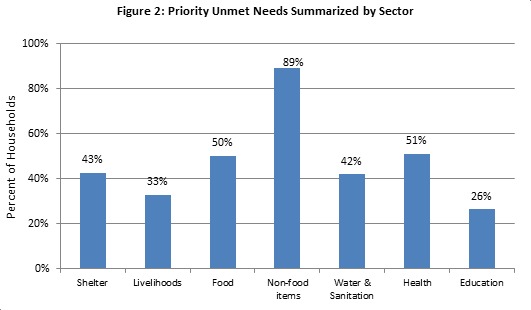

Approximately half (47.9%, CI: 44.8-51.1) of households received medical care in the month preceding the assessment. The primary reasons for not receiving expected care included out of stock medications (30%), facilities were too busy (21%), nearby facilities were unable to treat the condition (15%), health workers were not present (9%), cost (8%), the facility was destroyed (6%) and the facility was closed or occupied (5%).[2] Among households needing medication in the past month, the majority (77.8%, CI:74.3-79.8) were able to purchase medicines, however, 57.8% (CI: 55.4-60.1) reported difficulties in purchasing medications; 17.8% (CI: 16.1-19.7) were unable to purchase needed medications. Significant differences in purchasing of medications were observed across provinces (Annex III). Among those reporting being unable to purchase medicines or having difficulty purchasing medicines, the primary reasons included cost (80.4%, CI: 78.1-82.6) and out of stock medications (15.7%, CI: 13.8-17.9). To better understand the health needs, households were asked to identify members that required follow up or specialized care. Overall, 60.3% (CI: 58.1-62.4) of households had members that required specialized care; the most common reasons included chronic disease (37.2%), injury or disability (24.2%), older adult with illness (22.8%), and pregnant/lactating women (18.0%).†

High food prices were a critical concern that was nearly universal. The majority of households (73.1%, CI: 71.0-75.0) reported spending three-quarters or more of household income on food. Of the 89.6% (CI: 88.1-90.9) of households that reported difficulty in accessing food, 96.9% reported high prices as an obstacle; other barriers included insecurity (9.9%), unavailability of food in shops (8.5%) and distance (7.0%).† Overall, 10.9% (CI: 9.5-12.3) of households consumed one or fewer meals per day, an indication of extreme distress; 59.2%(CI: 57.0-61.4) consumed two meals per day, and 29.9% (CI: 27.9-32.0) three or more meals per day.

While education was not identified as an area of priority need by respondents, non-attendance rates are indicative of a serious disruption in the education of Syria’s youth. Of participating households, 82.7% (CI: 81.0-84.3) had a school age child. Among households with school age children, nearly a quarter (22.3%, CI: 20.5-24.1) reported having one or more children not enrolled in school. Households were more likely not send older children to school, with non-attendance reported by 30.8%, (CI: 28.8-32.8) of households for the 12-18 year age group, compared to 14.6% (CI: 13.1-16.2) of children in the 5-11 year age group. Most children had been out of school for extended lengths of time, with nearly half (47.1%, CI: 42.7-51.1) not attending for a year or more. Primary reasons for non-attendance included cost (43.5%) and insecurity (36.4%).†

‡ Confidence intervals not reported because percentage reflects aggregation across multiple categories

† Confidence intervals not reported because multiple responses were permitted

## Discussion

Despite receipt of ongoing assistance, almost all households reported unmet NFI needs and half of households reported unmet food needs. Among those reporting difficulty accessing food, high prices was the predominate reason, suggesting that food is available in markets but that households are having a difficult time accessing it due to inflation and decreasing incomes, otherwise known as entitlement failure.^3^ ^4^ The effects of the conflict on the Syrian economy and the livelihoods of many households, including both displaced and non-displaced, has been devastating and many families have lost regular income sources and are now forced to rely increasingly on informal and intermittent income sources. Humanitarian assistance interventions should focus on market strategies aimed at price reduction and also on targeting the most food insecure households, notably those consuming one or fewer meals per day, those without regular income sources and those that meet other vulnerability criteria such as high dependency ratios, female headed households or households with members that fall into vulnerable groups that are commonly targeted for additional assistance such as pregnant and lactating women or individuals with disabilities. While assistance modalities that support markets, such as cash transfer programs, may not be feasible or appropriate in all areas, in which case direct distribution of in-kind aid remains the only alternative. Maintaining access to affected populations to effectively target and implemented sustained humanitarian assistance is a major challenge in the context of the rapidly evolving Syrian conflict. While food security has clearly deteriorated compared to prior to the conflict and all indications are that the situation will continue to deteriorate, widespread increases in prevalence of acute malnutrition rates have not been reported. Anecdotal reports of increases in acute malnutrition in vulnerable populations and besieged areas are emerging,^5^ suggesting that community screening programs for children under-five could be a successful means of both identifying cases in areas with the highest levels of food insecurity and evaluating change in the nutrition situation and food security situation over time. Systematic and widespread monitoring of market prices, which is currently be undertaken by several humanitarian agencies in selected geographic areas, and broader synthesis of price trends would also be useful in understanding food access.

Another area of concern was the inability to, or difficulty in, accessing medications reported by many households, in large part because of high costs. Findings suggest that availability of medications was not a major concern, which is indication that markets and supply chains are still functioning to certain extent, at least in the private sector. Trends associated with the conflict more broadly, including declining capacity of the health system to provide health services and decreases in the availability of free and subsidized medications at public sector facilities are major concerns. This translates to reduced health service access and coverage, which is highly variable by location and conflict intensity, and reduced access to medication among lower income households, which will is likely to be more universal across areas of Syria. Supporting existing health infrastructure, in particular the NGO and charity sector, is one feasible approach to maintain health service access. Mobile clinics could serve as an interim means of increasing health service access in areas with high levels of unmet need and where security permits until more sustained health system reconstruction efforts can be undertaken during the post-conflict transition. With respect to efforts to ensure access to medications, identification and support to households whose members require routine medications that are expensive, in particular for chronic diseases, would contribute to improved management of these conditions and better health outcomes. Direct provision of medications, vouchers or cash subsidies to cover all or part of medication costs are potential strategies that could improve access.

The primary reasons reported for children not attending school were cost and insecurity. Insecurity is not an issue that easily addressed by humanitarian response organizations and it is likely that the areas with high non-attendance rates are also those that are the most insecure. However, given that cost-related reasons were the primary reason for non-attendance, some gains could be through programs that address these barriers. Conditional cash transfers or material support of school supplies may be effective means of helping households cover the costs of transportation and school fees and provision of school supplies, which could help to maintain school attendance and encourage re-enrollment.

Assessment findings suggest that interventions that increase access to non-food items, food, medication and education should be prioritized where cost was the primary barrier to accessing goods and services. Both cash transfer programs and direct provision of material assistance should be considered, although the most appropriate assistance modality is likely to vary by sector, location and related security and access considerations, and the preferences and prior experience of donors and implementing organizations. While the humanitarian response should be aligned with the needs of long-termed displaced and conflict-affected communities, well-targeted short-term emergency responses are also critical, in particular in areas of heavy conflict where both unmet needs and disruptions in access to basic services are likely to be the greatest. Findings from the needs assessment are a strong indication of the widespread unmet needs in Syria. However, they under represent the severity of the crisis and the extent of actual humanitarian needs. The limited number of the assessment teams in certain areas like Aleppo and Homs and the inability of the teams to access areas closer to the conflict lines for personal safety reasons, including regions that are not under government control, which have lower access levels to basic services and humanitarian assistance, is an important limitation of the study and its results. Taken in conjunction with the recent escalation of violence as the international community scales up their engagement in Syria, the magnitude of the humanitarian emergency is both under-estimated and likely to escalate. A renewed international commitment to funding humanitarian assistance efforts both with in Syria and among neighboring countries where the burden of refugees is greatest is essential from both a human rights perspective and in terms of maintaining stability in the region.

## Competing Interests

The authors have declared that no competing interests exist.
